# Assessing the use of finite element analysis for mechanical performance evaluation of intervertebral body fusion devices

**DOI:** 10.1002/jsp2.1137

**Published:** 2021-01-13

**Authors:** Andrew P. Baumann, Thomas Graf, Jonathan H. Peck, Anton E. Dmitriev, Dezba Coughlan, Jeffrey C. Lotz

**Affiliations:** ^1^ Office of Science and Engineering Laboratories, Center for Devices and Radiological Health U.S. Food and Drug Administration Silver Spring Maryland USA; ^2^ Office of Product Evaluation and Quality, Center for Devices and Radiological Health U.S. Food and Drug Administration Silver Spring Maryland USA; ^3^ Department of Orthopaedic Surgery University of California, San Francisco San Francisco California USA

**Keywords:** finite element analysis, intervertebral body fusion device, modeling, simulation

## Abstract

**Background:**

Intervertebral body fusion devices (IBFDs) are a widely used type of spinal implant placed between two vertebral bodies to stabilize the spine for fusion in the treatment of spinal pathologies. Assessing mechanical performance of these devices is critical during the design, verification, and regulatory evaluation phases of development. While traditionally evaluated with physical bench testing, empirical assessments are at times supplemented with computational models and simulations such as finite element analysis (FEA). However, unlike many mechanical bench tests, FEA lacks standardized practices and consistency of implementation.

**Objectives:**

The objectives of this study were twofold. First, to identify IBFD 510(k) submissions containing FEA and conduct a comprehensive review of the elements provided in the FEA reports. Second, to engage with spinal device manufacturers through an anonymous survey and assess their practices for implementing FEA.

**Methods:**

First, a retrospective analysis of 510(k) submissions for IBFDs cleared by the FDA between 2013 and 2017 was performed. The contents of FEA test reports were quantified according to FDA guidance. Second, a survey inquiring about the use of FEA was distributed to industry and academic stakeholders. The survey asked up to 20 questions relating to modeler experience and modeling practices.

**Results:**

Significant gaps were present in model test reports that deemed the data unreliable and, therefore, unusable for regulatory decision‐making in a high percentage of submissions. Nonetheless, the industry survey revealed most stakeholders employ FEA during device evaluation and are interested in more prescriptive guidelines for executing IBFD models.

**Conclusions:**

This study showed that while inconsistencies and gaps in FEA execution do exist within the spinal device community, the stakeholders are eager to work together in developing standardized approaches for executing computational models to support mechanical performance assessment of spinal devices in regulatory submissions.

## Graphical abstract

This manuscript describes an investigation of finite element analysis (FEA) practices for demonstrating mechanical performance of intervertebral spinal fusion devices. The manuscript reveals inconsistencies and gaps in FEA execution within the spinal device community and proposes that future efforts among stakeholders could be towards developing standardized approaches for executing computational models to support mechanical performance assessment of spinal devices in regulatory submissions.

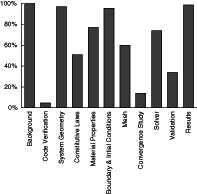

## 1 INTRODUCTION

Intervertebral body fusion devices (IBFDs) are a widely used type of spinal implant placed between two vertebral bodies to stabilize the spine for fusion in the treatment of spinal pathologies such as disc degeneration. IBFDs are available in a wide range of sizes, shapes, styles, and materials to match patient needs and surgeon preferences. After excising the intervertebral disc, an IBFD filled with bone graft material is placed between the vertebrae to stabilize the adjacent bones and maintain spacing during fusion. Prior to complete fusion, the device must sustain the complex loading conditions of the spine including compression, shear, torsion, bending, and combinations thereof. Adequate mechanical performance of these devices is important for maintaining safety and effective surgical outcomes for patients.

Spinal device manufacturers test their implants to consensus standards developed by standards development organizations (SDO). Appropriate standards are incorporated by the U.S. Food and Drug Administration (FDA) into guidance documents that articulate the Agency's perspective on appropriate testing regimens for a specific device class.^1^, ^2^ IBFDs are tested according to ASTM F2077, which prescribes compression, compression‐shear, and torsional testing in both static and fatigue modes.^3^ These are physical tests, meaning they must be performed “on the bench.” Bench testing is generally considered the gold standard for evaluating spinal cage mechanical performance. However, while bench testing will likely remain an important element in the IBFD design process, it is not the only way to characterize mechanical performance.

Computational modeling like finite element analysis (FEA) can also be used to simulate IBFD loading in silico. Computational frameworks can facilitate rapid testing of multiple designs under numerous loading scenarios. Moreover, FEA allows researchers to investigate localized phenomena such as stress concentrations that may be prohibitively difficult to observe with bench testing. Last, FEA is nondestructive. Different FEA models can be run without having to manufacture and destroy new test specimens. However, even with these advantages, implementing FEA in a robust fashion remains difficult. Few guidelines exist to help align modeling practices across stakeholder groups.

As with bench testing, having best practices for executing computational analyses helps to ensure credibility and comparability of results. Modeler expertise and availability of resources can lead to significant differences in model form, execution, and reporting of results. Documents have been developed to help guide FEA and subsequent reporting, such as the FDA guidance on Reporting of Computational Modeling Studies in Medical Device Submissions, ASTM International's standards, and the American Society of Mechanical Engineering (ASME) Verification and Validation (V&V) documents.^4^, ^5^, ^6^, ^7^, ^8^, ^9^ However, no such standard exists specifically for IBFDs, and implementation of FEA remains at the discretion of the modeler. Thus, it is important to define a best practices approach for FEA of IBFDs to help standardize numerical techniques and advance the use of FEA in the spinal device industry.

In order to define best practices for using FEA to evaluate mechanical performance of IBFDs, it is important to first understand the current state of simulations as well as the needs of stakeholders. Therefore, the objectives of this current study were 2‐fold. First, to identify IBFD 510(k) submissions containing FEA and conduct a comprehensive review of the reporting elements provided in the FEA reports. Second, to engage with spinal device manufacturers through an anonymous survey and assess their practices for implementing FEA in research and development activities. Together, these tasks would identify gaps in how FEA is currently being used and guide future efforts in defining best practices.

## 2 METHODS

### 2.1 510(k) review

Previously, a retrospective analysis of 510(k) submissions was conducted and mechanical performance of FDA‐cleared IBFDs were summarized.^10^, ^11^ In the current study, retrospective analysis of 510(k) submissions for IBFDs cleared by the FDA between 2013 and 2017 was performed and submissions containing FEA test reports were identified for further investigation. The contents of FEA test reports were then quantified according to the FDA guidance document on Reporting of Computational Modeling Studies in Medical Device Submissions.^4^ All FEA reports were reviewed for inclusion of information which aligned with the sections of the guidance document. These were: code verification, system geometry, constitutive laws, material properties, boundary and initial conditions, mesh (expanded to include a convergence study), solver, validation, and results. Only the presence of information which conformed to these sections was evaluated. The quality of the information was not assessed. Results were quantified by calculating the percentage of submissions that contained information conforming to the sections outlined in the guidance document, with 100% indicating that all reports contained a specified section.

### 2.2 Stakeholder survey

A survey was drafted inquiring about use of FEA. Depending upon responses, the survey would ask up to 20 questions relating to modeler experience and modeling practices. Questions were multiple choice and short answer format. For multiple choice questions, selection of more than one answer was allowed. The Center for Disruptive Musculoskeletal Innovations (CDMI, a National Science Foundation Industry and University Cooperative Research Center [NSF IUCRC]) distributed the survey to industry and academic stakeholders. Results were collated and analyzed for trends which may indicate the needs of modelers trying to demonstrate preclinical mechanical performance of IBFDs.

## 3 RESULTS

### 3.1 510(k) review

**FIGURE 1:**
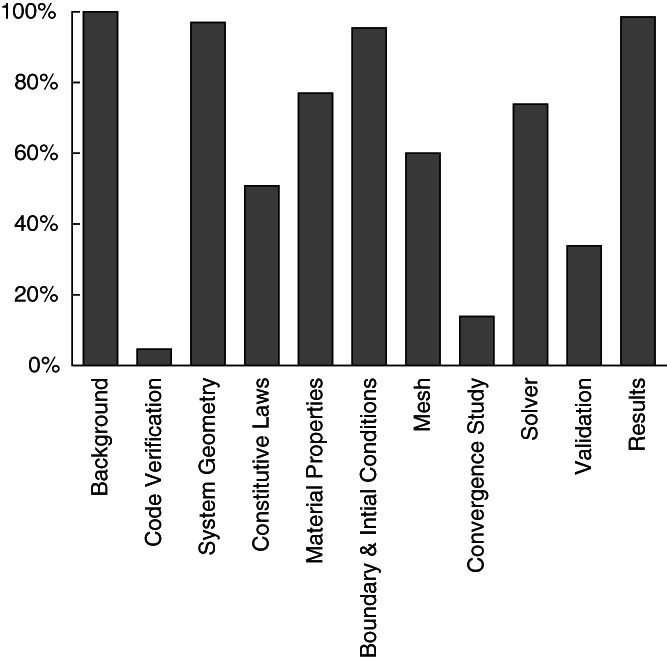
Percentages of the 510(k) submissions (n = 65) which contained information relating to the sections outlined in the FDA computational modeling reporting guidance document

Within the 5‐year timeline, a total of 65 premarket 510(k) submissions for IBFDs were identified which contained FEA test reports. Of those submissions, 100% contained background information, 5% contained code verification, 97% contained system geometry, 51% contained constitutive laws, 77% contained material properties, 95% contained boundary and initial conditions, 60% contained mesh information, 14% contained a convergence study, 74% contained solver information, 34% contained validation, and 98% contained results information (Figure 1).

General introduction and background language in the test reports indicated that all FEA was used to determine a worst‐case device size or shape to then be selected for bench testing according to ASTM F2077 (Table 1). Worst‐case selection was made by simulating the relevant loading modes outlined in ASTM F2077 on the range of IBFD designs in the 510(k) submission. Cage geometry was simplified more often than using the exact cage geometry. Load fixture geometry was included in 33 of the reports (51%), and most of these fixtures included pockets which mated with the cage geometry (35%). For constitutive laws, linear elasticity was used most often (42%). The most common loading and boundary conditions simulated compression testing (92%), followed by compression‐shear (49%), and torsion (34%). Among the 33 reports which included fixture geometry, 70% described contact conditions used between cages and fixtures. These included bonded (27%), no separation (24%), friction (12%), and frictionless (6%). The most frequently used validation activity was comparison to bench testing results (31%). In the results section, the most commonly reported outcome was von Mises stress (77%), with principal stress (5%) and unspecified stresses (17%) also being provided.

**TABLE 1:** Detailed quantification of information provided in FEA reports

Item	Fraction	Percent
Background		
Background description	65/65	100%
Code verification		
Verification activities	3/65	5%
System geometry		
Cage geometry	63/65	97%
Full cage	26/65	40%
Simplified cage	37/65	57%
Fixture geometry	33/65	51%
With pockets	23/65	35%
Without pockets	10/65	15%
Constitutive laws		
Constitutive law	33/65	51%
Linear	27/65	42%
Bilinear	2/65	3%
Nonlinear	4/65	6%
Material properties		
Cage	50/65	77%
Fixtures^a^	26/33	79%
Boundary and initial conditions		
Boundary and load conditions	62/65	95%
Compression	60/65	92%
Compression‐shear	32/65	49%
Torsion	22/65	34%
Contact conditions^a^	23/33	70%
Bonded	9/33	27%
No separation	8/33	24%
Friction	4/33	12%
Frictionless	2/33	6%
Mesh		
Mesh information	39/65	60%
Convergence study	9/65	14%
Solver		
Solver information	48/65	74%
Validation		
Validation activities	22/65	34%
Bench test comparator	20/65	31%
Other comparator	3/65	5%
Results		
Stress reported	64/65	98%
von Mises stress	50/65	77%
Principal stress	3/65	5%
Unspecified stress	11/65	17%

*Note:* Results are organized according to the sections described in the FDA computational modeling reporting guidance document. Major items are quantified as a Fraction and Percent of all identified 510(k) submissions (n = 65).

^a^ Fraction and percent calculated relative to the number of models containing fixture geometry (n = 33).

### 3.2 Stakeholder survey

A total of 31 stakeholders from within the United States participated in the survey (Table 2). All respondents affirmed employing FEA through internal resources or consulting services. This was most often done for research and development or prototyping (27/69, 39%) as well as regulatory needs (34/69, 49%). The choice of finite element software varied. Responses indicated that FEA most often has a medium influence in decision‐making (16/31, 52%). The majority of stakeholders self‐identified as intermediate (14/31, 45%) or expert (12/31, 39%) level modelers with the most common duration of modeling experience being 10 or more years (13/31, 42%).

**TABLE 2:** Stakeholder survey questions and accompanying results

1: Do you conduct finite element analysis (FEA)?			
	Answer	%	Count
	Yes	84%	26
	No	0%	0
	Outsource	16%	5
	Total	100%	31
2: How do you use FEA?			
	Answer	%	Count
	R&D/Prototyping	39%	27
	Regulatory ‐ determining worst case	23%	16
	Regulatory ‐ comparative analysis	26%	18
	Other, please add:	12%	8
	N/A (do not use FEA)	0%	0
	Total	100%	69
3: What FEA software do you use?			
	Answer	%	Count
	Abaqus	34%	17
	ADINA	0%	0
	ANSYS	28%	14
	Creo Simulate	2%	1
	LS‐DYNA	4%	2
	SolidWorks Simulation	20%	10
	Other, please add:	12%	6
	N/A (Do not use FEA)	0%	0
	Total	100%	50
4: How much influence does FEA have in your decision‐making?			
	Answer	%	Count
	None	0%	0
	Low	16%	5
	Medium	52%	16
	High	32%	10
	N/A (do not use FEA)	0%	0
	Total	100%	31
5: What is you experience level with FEA?			
	Answer	%	Count
	Novice	13%	4
	Intermediate	45%	14
	Expert	39%	12
	N/A (do not use FEA)	3%	1
	Total	100%	31
6: How long have you been doing FE simulations?			
	Answer	%	Count
	0‐2 y	16%	5
	3‐5 y	32%	10
	6‐9 y	10%	3
	10+ y	42%	13
	N/A (do not use FEA)	0%	0
	Total	100%	31
7: Do you consult any documents regarding FEA best‐practices?			
	Answer	%	Count
	Yes	42%	13
	No	26%	8
	No ‐ I was not aware of such documents	32%	10
	N/A (do not use FEA)	0%	0
	Total	100%	31
8: What guidance documents do you consult?			
	Answer	%	Count
	FDA Computational Modeling Guidance	59%	10
	ASTM F2996	6%	1
	ASTM F3161	0%	0
	ASME V&V 10	18%	3
	Other, please add:	18%	3
	Total	100%	17
9: What do you find most valuable from these documents?			
	13 total responses		
10: What do you find most limiting from these documents?			
	10 total responses		
11: What additional information/guidance is needed from these documents?			
	9 total responses		
12: If you consult multiple documents, do they provide consistent best‐practices?			
	Answer	%	Count
	Yes	33%	1
	No	67%	2
	Total	100%	3
13: Are you performing model validation?			
	Answer	%	Count
	Yes	90%	26
	No	10%	3
	N/A (do not use FEA)	0%	0
	Total	100%	29
14: What validation is performed?			
	Answer	%	Count
	Comparison to bench testing	36%	25
	Comparison to the literature	26%	18
	Comparison to previously validated FEA	20%	14
	Comparison to analytical solution	14%	10
	Other, please add:	3%	2
	Total	100%	69
15: Do you use an internal or external model validation document?			
	Answer	%	Count
	No	56%	18
	Internal ‐ please elaborate:	22%	7
	External ‐ please elaborate:	22%	7
	Total	100%	32
16: How do you define model material properties?			
	Answer	%	Count
	Internet search	30%	19
	Internal testing	28%	18
	Material database	33%	21
	Other, please add:	9%	6
	N/A (do not use FEA)	0%	0
	Total	100%	64
17: Would you find a FEA best‐practices document specific to spinal devices valuable?			
	Answer	%	Count
	Yes	86%	24
	No	14%	4
	Total	100%	28
18: What level of information would you like to see in the document?			
	Answer	%	Count
	Basic (general practices)	8%	2
	Intermediate (descriptive practices)	42%	10
	Advanced (fully prescribed practices)	46%	11
	Other, please add:	4%	1
	Total	100%	24
19: Would you like to have a group of experts dedicated to the use of FEA for cage design and development available to help your company?			
	Answer	%	Count
	Yes	61%	17
	No	39%	11
	Total	100%	28
20: List some of the qualifications you would like to see in that group.			
	16 total responses		

*Note:* The number of subjects that responded to a question are presented as a percentage (%) and count. Some questions allowed for multiple responses to be selected.

Most stakeholders did not consult any documents regarding FEA best‐practices (18/31, 58%), and approximately one‐third were not aware of any such documents (10/31, 32%). The best practices document most often referenced was the FDA Reporting of Computational Modeling Studies in Medical Device Submissions guidance document (10/17, 59%). Other documents identified with short answer included software manuals, forums, journal publications, internal guidance documents, and standards. Thirteen stakeholders provided items they found most valuable in these best‐practices documents. Some of these items included methods, boundary conditions, material properties, assistance communicating evidence in a consistent manner, validation, procedures, reporting, and others. Ten stakeholders also provided items they found limiting in these best‐practices documents. The most common item was some type of lack of specificity. This was expressed as incomplete, obscure, vagueness, generalized, and never detailed enough. These shortcomings were echoed when stakeholders indicated what was needed from best‐practices documents: full methods, less obscurity, specific regulations, and more examples.

Nearly all surveyed stakeholders were performing model validation activities (26/29, 90%) with the most common method being a comparison to bench testing (25/69, 36%). Fewer stakeholders were using the literature (18/69, 26%), previously validated FEA (14/69 20%), and analytical solutions (10/69, 14%) as validation comparators. Also, most stakeholders were not consulting an established validation protocol (18/32, 56%). Those that did use a validation document were split between internal (7/32, 22%) and external (7/32, 22%) documentation. Material properties were retrieved from a variety of sources with relatively equal distribution between internet searches (19/64, 30%), internal testing (18/64, 28%), and material databases (21/64, 33%).

When asked if they would find a best‐practices document specific to spinal devices valuable, 86% (24/28) of surveyed stakeholders indicated yes. Moreover, stakeholders showed that such a document would benefit from having intermediate (10/24, 42%) or advanced (11/24, 46%) levels of prescriptive information, as opposed to basic general practices (2/24, 8%). Most stakeholders also reported they would value having a group of FEA experts available to assist with device design and development (17/28, 61%). Sixteen stakeholders provided examples of the qualifications they would expect in this group, which included clinical experience, engineering experience, spinal biomechanics expertise, FEA experience, advanced degrees, familiarity with regulatory requirements, experimental mechanics experience, familiarity with standards, understanding verification and validation practices, and having practiced in their field for multiple years.

## 4 DISCUSSION

FEA can be a versatile tool for assessing the mechanical performance of IBFDs. However, unlike traditional bench testing which has longstanding standardized methods for testing and reporting, FEA has comparatively few guidelines for executing and communicating a study. Medical device manufacturers and regulatory officials understand the potential value of computational modeling in development of new devices.^12^ However, analysts must make many decisions when constructing a model and establishing model credibility.^13^, ^14^ Their choices are often influenced by education, expertise, and resources. Moreover, modeler choices will often have a significant effect on simulation outcomes. Establishing best practices for executing FEA for IBFDs will help harmonize methods across stakeholder groups, leading to more repeatable modeling practices, greater credibility in results, and more consistent reporting. The current study served as a first step in this direction by increasing the understanding of the current landscape of FEA use and model quality presented to the FDA as part of IBFD 510(k) submissions. Additionally, useful real‐time feedback was obtained from industry stakeholders on their current modeling practices and modeler needs.

Results of the FDA 510(k) submission review showed large disparities in the information provided in FEA reports. Some sections like system geometry, boundary and initial conditions, and results were nearly always reported. Conversely, other sections such as code verification, validation, and a mesh convergence study were provided no more than one third of the time. In order for regulators to establish credibility in computational models, all sections should be adequately represented. While most of the recommended information may be present in a FEA report, the information is only useful if the report contains all sections. Therefore, it is critical for regulators to clearly articulate expectations, and for stakeholders to adhere to them. Failure to do so may produce FEA results that are unreliable or difficult to interpret, which may in turn undermine the overall utility of the model for the purposes of regulatory review and decision making.

The 510(k) review also revealed that modelers are choosing to simulate mechanical performance testing in many different ways. In all cases, the simulations were conducted for the purposes of identifying a worst‐case device(s) for subsequent bench testing per ASTM F2077. Moreover, all reports indicated that models were based on physical testing prescribed by ASTM F2077. Despite the high level of alignment in the purpose and physical comparator of the FEA studies, the resulting analyses took on many different forms. Most models simulated compression, but many modelers also simulated compression‐shear as well as torsion. In order to obtain consistent results, stakeholders will have to agree upon the appropriate methods for applying loading and boundary conditions for each of these loading scenarios. For example, replicating ASTM F2077 bench tests may necessitate inclusion of load fixtures. Replicating load application through boundary conditions may overly simplify the model and produce results that do not represent the bench test comparator. Simulating fixtures, though, adds complexity and requires additional choices. Modelers must decide whether to include pockets within the platens, how deep to make them, and how to model the resulting contact between surfaces (rigid bonding, friction law, etc). Modelers were also split as to how to model the IBFD geometry, with roughly half simulating the exact geometry, while the other half simplified the geometry. Simulating exact geometry may be problematic due to sharp features such as teeth or threads. These features may create reentrant corners which can introduce local singularities and artificially high stress/strain concentrations that confound results.^15^, ^16^, ^17^ Therefore, cautious removal of such features or exclusion of results in the immediate area may be appropriate. However, certain simplifications may remove critical features that affect model outcomes. While singularities can cloud modeling results, they can also point to problematic geometry which may lead to device failure. Without accepted practices, geometries will be simplified in different ways, with some simplifications being acceptable and some potentially leading to erroneous results and false conclusions. All these choices can influence results. Developing guidelines will help modelers perform simulations in a more consistent and repeatable fashion.

There was a large discrepancy between the 510(k) review and the stakeholder survey regarding model validation. Of the 65 submissions, only 34% contained validation of any kind. However, the stakeholder survey revealed that most are performing validation activities, with 90% claiming to validate their models. Reasons for this disparity are unclear. It is possible that some modelers are unaware or unfamiliar with validation. However, given that the survey returned nearly unanimous execution of validation exercises, it is also possible that modelers are simply not reporting these activities to FDA. Additionally, the survey participants may have represented a significantly different population than those companies which had submitted the reviewed 510(k)s. The stakeholder survey was distributed to well established institutions that have mature validation and reporting practices. Conversely, the reviewed 510(k) submissions may have come from companies with a wide range of modeling expertise. Some of these may have represented small startup organizations with limited validation experience. Educational outreach may help ensure all organizations can provide necessary information in device submissions. Therefore, FDA must continue to make its reporting expectations clear, and stress that inclusion of validation activities is important for regulatory review of computational models.

A limitation of this study stems from using the FDA computational modeling reporting guidance sections as criteria for assessing the 510(k) submissions. The final guidance document was issued on 21 September 2016. The reviewed 510(k) submissions spanned this issuance, ranging from 2013 to 2017. As such, 510(k)s predating the guidance document would not have been prepared to align with its reporting expectations. Despite this, most sections are included with several exceeding a 90% reporting rate. Reports may include this information because the sections of interest are often articulated in other sources. Modelers with a formal education in FEA are generally exposed to these principles in textbooks.^18^, ^19^ Additionally, these sections are described to varying degrees in ASME V&V 10,^7^ NASA‐STD‐7009,^20^ SAND2007‐5948,^21^ and Department of Defense verification and validation References 22, 23. Modelers with greater levels of experience may be better informed about the existence of these documents and the recommendations for all FEA reports contained therein. Therefore, despite the FEA computational modeling reporting guidance being published midway through the analysis period, it is not unreasonable to expect FEA reports to contain many of the elements recommended in this guidance.

## 5 CONCLUSION

IBFD stakeholders recognize the potential value of FEA in the assessment of device mechanical performance. Review of current FEA use in 510(k) marketing applications submitted to the FDA for IBFDs revealed major gaps and inconsistencies in model reporting. The subsequent industry survey, however, identified that FEA is used by most surveyed stakeholders with the majority stating that FEA has at least a medium influence in both early development as well as final testing to satisfy regulatory requirements. Moreover, the survey indicated that more prescriptive technical considerations for executing IBFD FEA would be advantageous. These findings highlight the need for the spinal device community to develop best practices for performing computational modeling and develop relevant consensus standards. Additional educational efforts are also warranted to ensure these approaches are clearly communicated and well‐understood by all stakeholders. These steps will improve our confidence in modeling and simulation and enhance its role in regulatory review, thereby reducing costs and burden for the community.

## CONFLICT OF INTEREST

The authors declare no potential conflict of interest.

## AUTHOR CONTRIBUTIONS

All authors have provided substantial contributions to research design, or the acquisition, analysis or interpretation of data, contributed to drafting the article or revising it critically, and provided approval of the submitted and final versions. All authors have read and approved the final submitted manuscript.
